# Vascular endothelial cadherin shedding is more severe in sepsis patients with severe acute kidney injury

**DOI:** 10.1186/s13054-019-2315-y

**Published:** 2019-01-18

**Authors:** Wen-Kuang Yu, J. Brennan McNeil, Nancy E. Wickersham, Ciara M. Shaver, Julie A. Bastarache, Lorraine B. Ware

**Affiliations:** 1 Division of Respiratory Therapy, Department of Chest Medicine, Taipei Veterans General Hospital, No. 201, Sec. 2, Shipai Rd., Beitou District, Taipei City, 11217 Taiwan, Republic of China; 2 Institute of Physiology, National Yang-Ming University, Taipei, Taiwan; 3 Division of Allergy, Pulmonary, and Critical Care Medicine, Department of Medicine, Vanderbilt University Medical Center, T1218 MCN, 1161 21st, Avenue S, Nashville, TN 37232 USA; 4 Department of Cell and Developmental Biology, Vanderbilt University School of Medicine, Nashville, TN USA; 5 Department of Pathology, Microbiology and Immunology, Vanderbilt University School of Medicine, Nashville, TN USA

**Keywords:** Endothelial injury, Sepsis, Soluble vascular endothelial cadherin, Acute kidney injury, Renal replacement therapy

## Abstract

**Background:**

Vascular endothelial cadherin (VE-cadherin) is a membrane protein that is the major component of adherens junctions between endothelial cells. It is crucial for regulating vascular integrity, endothelial permeability, and angiogenesis. During inflammatory processes, VE-cadherin is shed into circulation (sVE-cadherin). Plasma sVE-cadherin is elevated in sepsis, malignancy, autoimmune diseases, and coronary atherosclerosis. However, the relationship between specific organ failures, especially severe acute kidney injury (AKI) defined by requirement for renal replacement therapy (AKI-RRT), and plasma sVE-cadherin levels in severe sepsis has not been well studied.

**Methods:**

The present study is a prospective study of critically ill adults with sepsis and acute respiratory failure (age ≥ 18 years) enrolled in the Validating Acute Lung Injury markers for Diagnosis (VALID) study. Plasma sVE-cadherin was measured at study enrollment. Primary analysis focused on the association between sVE-cadherin levels and the development of AKI, AKI-RRT, other organ dysfunction as defined by Brussels organ failure scores, pulmonary versus non-pulmonary sepsis, acute respiratory distress syndrome (ARDS), and in-hospital mortality.

**Results:**

Of 228 severe sepsis patients included, 80 (35%) developed AKI-RRT. Plasma sVE-cadherin levels at enrollment were significantly higher in patients with AKI-RRT compared with patients without AKI-RRT (*p* = 0.003). Plasma sVE-cadherin levels by quartile were significantly higher in severe sepsis patients with acute kidney injury stage 3 (*p* = 0.044) as defined by Kidney Disease Improving Global Outcomes (KDIGO) criteria. Patients with greater than 2 organ failures had higher plasma sVE-cadherin levels than patients with 2 or fewer organ failures (*p* < 0.001). In a multivariable analysis, plasma sVE-cadherin was independently associated with AKI-RRT (odds ratio 6.44 per log increase in plasma sVE-cadherin, 95% CI 1.126–36.847, *p* = 0.036). Plasma sVE-cadherin levels were significantly higher in patients with non-pulmonary sepsis compared to pulmonary sepsis (*p* < 0.001).

**Conclusion:**

Shedding of sVE-cadherin is associated with severe acute kidney injury and with more severe organ dysfunction in patients with sepsis, suggesting that breakdown of endothelial adherens junctions may contribute to the pathogenesis of organ dysfunction in sepsis. Further studies of sVE-cadherin as a biomarker of disease severity in clinical sepsis are needed to better elucidate the role of VE-cadherin shedding in sepsis-induced severe organ dysfunction.

## Introduction

Despite aggressive supportive treatment and prompt antibiotic administration, sepsis remains a life-threatening complication of infection [1–4]. The endothelium plays a vital role in the pathogenesis of organ dysfunction in sepsis. Endothelial injury and dysfunction lead to the breakdown of the microvascular barrier resulting in increased extravascular fluid, tissue edema, organ dysfunction, and death [5–8].

The endothelial barrier is composed of tight and adherens junctions. Vascular endothelial cadherin (VE-cadherin), an endothelial transmembrane glycoprotein, is the major cell-cell adhesion molecule that forms adherens junctions [9, 10]. During inflammatory processes, the extracellular domain of VE-cadherin can be cleaved by neutrophil elastase and specific disintegrins and metalloproteinases (ADAMs) and shed into the circulation as soluble VE-cadherin (sVE-cadherin) [11–13]. Several human studies showed elevated serum levels of sVE-cadherin in disorders associated with endothelial dysfunction including systemic vasculitis, malignancy, rheumatoid arthritis, and coronary atherosclerosis [14–18]. Plasma levels of sVE-cadherin are also elevated in sepsis patients, and the degree of shedding of VE-cadherin is correlated with the severity of sepsis and poor outcomes [19]. However, the relationship between specific organ failures and sVE-cadherin level in sepsis has not been studied.

Acute kidney injury (AKI) is a sudden decrease in renal function, characterized by an increase in serum creatinine level or decrease in urine output that results in significant morbidity and mortality, especially if renal replacement therapy is required (AKI-RRT) [20–22]. There are many causes of AKI during critical illness, including sepsis, trauma, shock, surgery, and nephrotoxic agents [23]. Although the role of tubular epithelial injury in AKI has been well studied, the contribution of endothelial injury is less well understood [24, 25]. A previous report demonstrated plasma sVE-cadherin levels were increased in patients on chronic hemodialysis [26]. However, whether or not shedding of VE-cadherin is associated with AKI in sepsis patients is not known. To test the hypothesis that VE-cadherin shedding is associated with sepsis-related AKI-RRT, we measured plasma sVE-cadherin levels in sepsis patients with or without AKI-RRT. We also evaluated the relationship between the degree of VE-cadherin shedding and other organ dysfunction.

## Methods

### Study design and patient selection

This was a prospective study of critically ill adults (age ≥ 18 years) who were enrolled in the Validating Acute Lung Injury markers for Diagnosis (VALID) study [27, 28]. VALID is a single-center prospective observational cohort study, which has enrolled critically ill patients admitted to Vanderbilt Medical, Surgical, Trauma and Cardiovascular ICUs since 2006. Patients were enrolled in VALID on the morning of ICU day 2. Patients were excluded from VALID if they had cardiac arrest prior to enrollment, drug overdose, and chronic lung disease requiring home oxygen therapy or did not remain in the ICU for at least 2 days. The study protocol was approved by Vanderbilt Institutional Review Board (IRB #051065). Informed consent was obtained for sample collection from the patients or surrogates whenever possible; if patients or surrogates were unable to provide consent, the institutional review board granted a waiver of consent due to the minimal risk nature of the study. At the time of enrollment, clinical data including patient demographics, medical history, prehospital medications, admission diagnoses, and acute physiology and chronic health evaluation II (APACHE II) [29] were collected. Laboratory values, hemodynamic variables, ventilator settings, in-hospital medications, fluid balance, AKI, acute respiratory distress syndrome (ARDS), and evidence of organ failures according to Brussels organ failure scores [30] were recorded daily through the morning of ICU day 5. Clinical outcomes including duration of mechanical ventilation and hospital mortality were also collected.

To enrich for patients with a high likelihood of AKI, the present study included mechanically ventilated medical ICU patients enrolled in the VALID study who had severe sepsis according to the sepsis-2 criteria [31] and APACHE II scores greater than 25. Patients with known end-stage renal disease at study enrollment were excluded. Patients with pneumonia or aspiration of gastric contents were grouped as pulmonary sepsis, and the remaining patients were grouped as non-pulmonary sepsis. Patients included in the current study were also included in a prior study of syndecan-1 levels in severe sepsis [27].

### Definition of AKI, chronic kidney disease, acute respiratory distress syndrome (ARDS), and non-pulmonary organ failure

Definition and stage of AKI were determined using serum creatinine and urine output according to the Kidney Disease Improving Global Outcomes (KDIGO) criteria during the study period [23]. Briefly, AKI was defined as an increase in serum creatinine ≥ 0.3 mg/dl or ≥ 50% within 48 h, or urine output less than for 0.5 ml/kg/h for at least 6 h. The criteria for AKI stage 3 included a serum creatinine level three times greater than the baseline, an increase in serum creatinine to ≥ 4.0 mg/dl, initiation of renal replacement therapy, a decrease in urine output to less than 0.3 ml/kg/h for ≥ 24 h, or anuria for ≥ 12 h [23]. Diagnosis of chronic kidney disease was based on the medical record. ARDS was assessed daily according to the Berlin definition, based on chest radiographs, blood gases, and clinical data by two physician investigators [32]. SpO2/FiO2 ratio was substituted for PaO2/FiO2 ratio for the diagnosis of ARDS if blood gas data were not available [33, 34]. Definition of circulatory, coagulation, and hepatic failure were based on the Brussels organ failure scores [30].

### Measurement of sVE-cadherin

Plasma levels of sVE-cadherin were measured in duplicate in citrated plasma samples collected at the time of study enrollment using enzyme-linked immunosorbent assay kits (R&D Systems, Minneapolis, MN, USA) according to the manufacturer’s instructions.

### Statistical analysis

Categorical variables were reported as counts and percentages and are analyzed by chi-square test. Continuous variables with normal distribution were presented as means with standard deviation and analyzed by independent samples *T* test. Continuous variables that were not normally distributed are presented as median with interquartile range and were analyzed by Mann-Whitney *U* test or Kruskal-Wallis *H* test. The association between sVE-cadherin quartile and categorical outcomes of interest was analyzed by linear-by-linear association test. To determine whether sVE-cadherin levels were independently associated with severe AKI, sVE-cadherin levels were log transformed and multivariable logistic regression was performed. Known predictors of AKI-RRT including chronic kidney disease, vasopressor use, and APACHE II score were included as covariates. A *p* value < 0.05 was considered to be significant. All analyses were performed using SPSS statistics version 19.0 (IBM, Armonk, NY).

## Results

### Patient characteristics

Two hundred twenty-eight critically ill patients with severe sepsis requiring invasive mechanical ventilator support were included in the study. Their demographic and clinical parameters are listed in Table 1. Among these patients, 80 (35%) developed AKI-RRT. Patients with AKI-RRT had a higher serum creatinine level at enrollment (*p* < 0.001), a longer ICU length of stay (*p* = 0.020), a longer hospital length stay (*p* = 0.013), fewer ventilator-free days (*p* = 0.001), higher APACHE II scores (*p* < 0.001), and a higher hospital mortality rate (*p* = 0.014) compared with severe septic patients without AKI-RRT.

**Table 1:** Clinical characteristics of 228 critically ill medical ICU patients with severe sepsis

	All patients (*N* = 228)	With AKI-RRT (*N* = 80)	Without AKI-RRT (*N* = 148)	*p* value
Age (years)	55 ± 16	54 ± 14	56 ± 17	0.639
Male	117 (51)	45 (56)	72 (49)	0.331
APACHE II	32 [29–37]	35 [31–40]	31 [28–35]	< 0.001
Ever smoker	140 (61)	50 (63)	90 (61)	0.887
Alcohol abuse	56 (25)	19 (24)	37 (25)	0.873
Chronic kidney disease	37 (16)	18 (23)	19 (13)	0.063
Vasopressor use	128 (56)	47 (59)	81 (55)	0.579
ARDS development	111 (49)	41 (51)	70 (47)	0.582
Ventilator-free days	19 [0–24]	6 [0–23]	21 [1–25]	0.001
ICU days	8 [5–11]	9 [6–13]	7 [4–11]	0.020
Hospital days	13 [8–22]	16 [9–26]	11 [8–20]	0.013
Died in hospital	83 (36)	38 (48)	45 (30)	0.014
Creatinine (mg/dl)	1.43 [0.92–2.63]	1.98 [1.15–4.06]	1.21 [0.83–2.03]	< 0.001
sVE-cadherin (ng/ml)	2697 [2110–3492]	3052 [2341–3887]	2543 [2058–3410]	0.003

Continuous data are expressed as mean ± standard deviation (SD) or median with interquartile range [IQR] as indicated, and categorical data are expressed as number of patients (%). *p* value is analyzed by *T* test (age), chi-square (gender, smoking, alcohol history, chronic kidney disease, vasopressor use, ARDS development, and mortality), or Mann-Whitney *U* test (APACHE II score, ventilator-free days, ICU days, hospital days, and sVE-cadherin) as appropriate

*RRT* renal replacement therapy, *APACHE II* acute physiology and chronic health evaluation II, *ARDS* acute respiratory distress syndrome, *ICU* intensive care unit

### Plasma sVE-cadherin levels and acute kidney injury

**Fig. 1:**
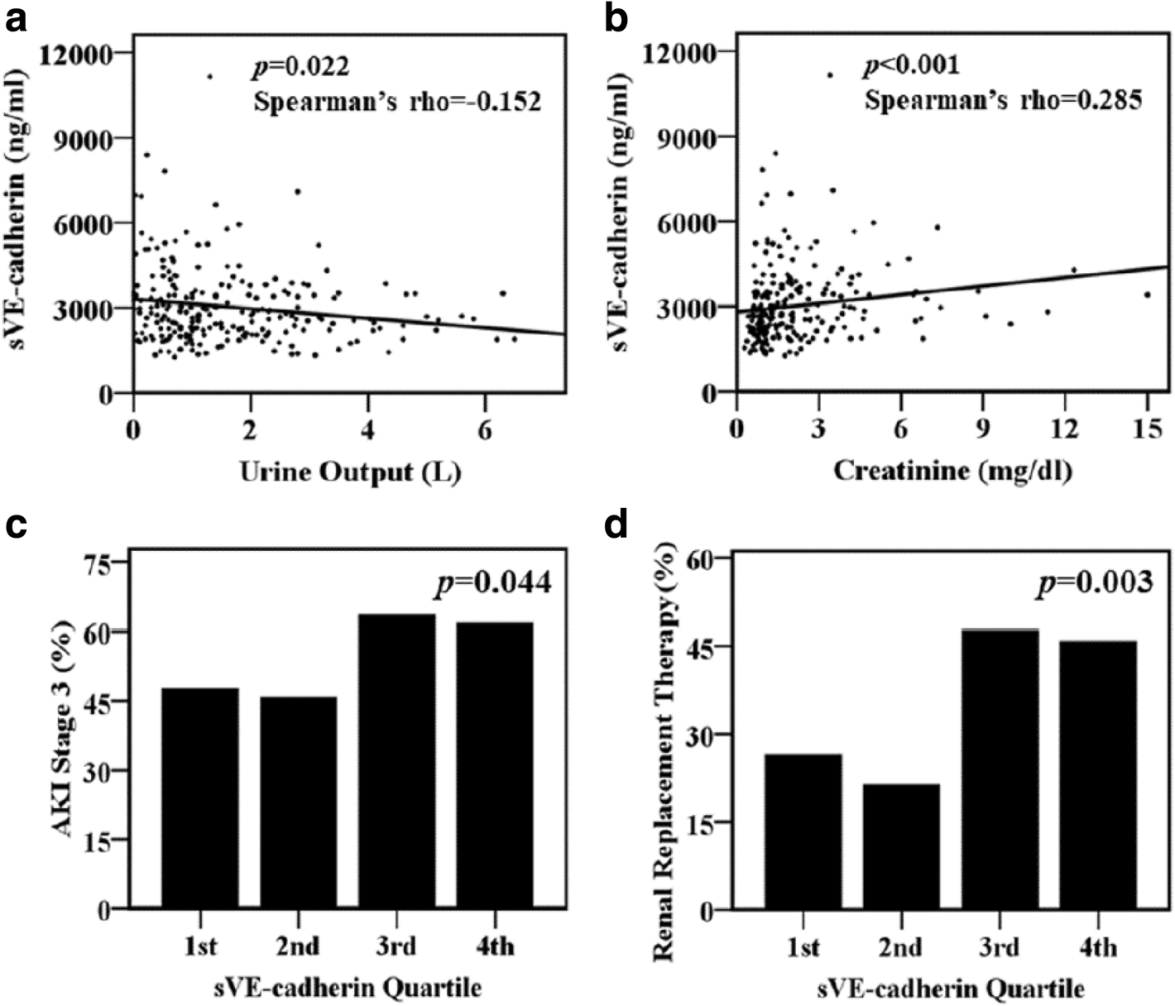
Plasma sVE-cadherin levels were weakly associated with **a** urine output and **b** serum creatinine levels on enrolling day. Higher plasma sVE-cadherin levels by quartile were significantly associated with **c** AKI stage 3 and **d** the need for renal replacement therapy

To evaluate the relationship between acute kidney injury and plasma sVE-cadherin level, urine output and serum creatinine were measured for 4 consecutive days after ICU admission. Higher plasma sVE-cadherin levels on enrolling day were weakly associated with lower daily urine output (Spearman’s rho − 0.152, *p* = 0.022, Fig. 1a) and higher serum creatinine levels (Spearman’s rho 0.285, *p* < 0.001, Fig. 1b) on enrolling day. Plasma sVE-cadherin levels were more strongly associated with more severe AKI. Levels were significantly higher in severe septic patients with AKI-RRT (3052 ng/ml, IQR 2341–3887 ng/ml vs. 2543 ng/ml, IQR 2058–3410 ng/ml, *p* = 0.003) compared with septic patients without AKI-RRT (Table 1). 89.9% of severe sepsis patients developed AKI within the first 2 days after enrollment. Among patients with normal renal function at enrollment, the plasma levels of sVE-cadherin at enrollment were not associated with new onset of AKI on the third or fourth ICU day (data not shown). When plasma sVE-cadherin levels were grouped by quartile, patients in the higher plasma sVE-cadherin quartiles were more likely to have AKI stage 3 (*p* = 0.044, Fig. 1c) and AKI-RRT (*p* = 0.003, Fig. 1d). Higher plasma sVE-cadherin levels were also independently associated with AKI-RRT (odds ratio 6.44 per log increase in plasma sVE-cadherin, 95% CI 1.126–36.847, *p* = 0.036, Table 2) in a multivariable logistic regression model adjusted for APACHE II score, vasopressor use, and chronic kidney disease.

**Table 2:** Multivariable logistic regression model for AKI-RRT in 228 septic patients

Variables	OR (95% CI)	*p* value
APACHE II (per one point increase)	1.095 (1.042–1.151)	< 0.001
Chronic kidney disease	0.611 (0.286–1.303)	0.202
Vasopressor use	1.069 (0.593–1.925)	0.825
sVE-cadherin (per log increase)	6.440 (1.126–36.847)	0.036

Chronic kidney disease is according to the medical records

### Plasma sVE-cadherin levels and other organ failures

**Fig. 2:**
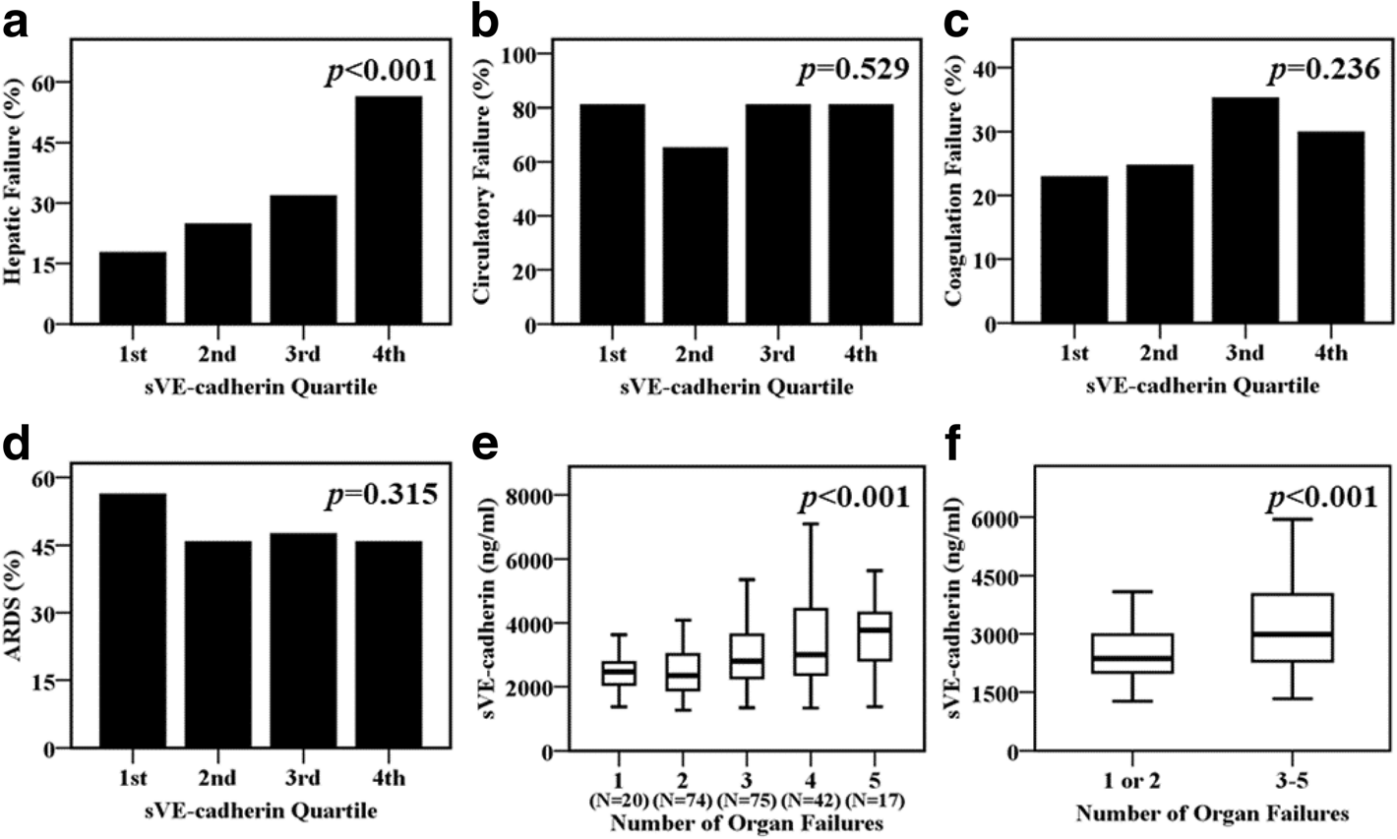
Higher plasma sVE-cadherin levels by quartile were associated with **a** hepatic failure on enrolling day, but not with **b** circulatory failure on enrolling day or **c** coagulation failure on enrolling day. Organ failures were defined by Brussels organ failure scores [30]. **d** Plasma sVE-cadherin levels were not associated with the development of ARDS defined by Berlin criteria during the study period [32]. Data in panels **a**–**d** were analyzed by linear-by-linear association test. **e** Plasma sVE-cadherin levels were associated with the number of organ failures on enrolling day. **f** Plasma sVE-cadherin levels were significantly higher in patients with greater than 2 organ failures compared with patients with 2 or fewer organ failures on enrolling day. Data in panel **e** and **f** were summarized as boxplots where box encompasses 25th–75th percentile, error bars encompass 10th–90th percentile, and horizontal line shows median. Data in panels **e** and **f** were analyzed by Kruskal-Wallis *H* test

Higher plasma sVE-cadherin levels by quartile at study enrollment were significantly associated with hepatic failure on enrolling day (*p* < 0.001, Fig. 2a) as defined by Brussels organ failure scores. Plasma sVE-cadherin levels were not associated with circulatory failure on enrolling day (*p* = 0.529, Fig. 2b), coagulation failure on enrolling day (*p* = 0.236, Fig. 2c), or development of ARDS during the study period (*p* = 0.315, Fig. 2d). Plasma sVE-cadherin levels were significantly higher in subjects with a greater number of organ failures on enrolling day (*p* < 0.001, Fig. 2e). Patients with greater than 2 organ failures had higher plasma sVE-cadherin levels compared to those with 2 or fewer organ failures on enrolling day (2992 ng/ml, IQR 2293–4040 ng/ml vs. 2364 ng/ml, IQR 2006–2987 ng/ml, *p* < 0.001, Fig. 2f). Plasma sVE-cadherin levels were not associated with the later onset of new organ failures (data not shown).

### Plasma sVE-cadherin levels and mortality and etiology of sepsis

**Fig. 3:**
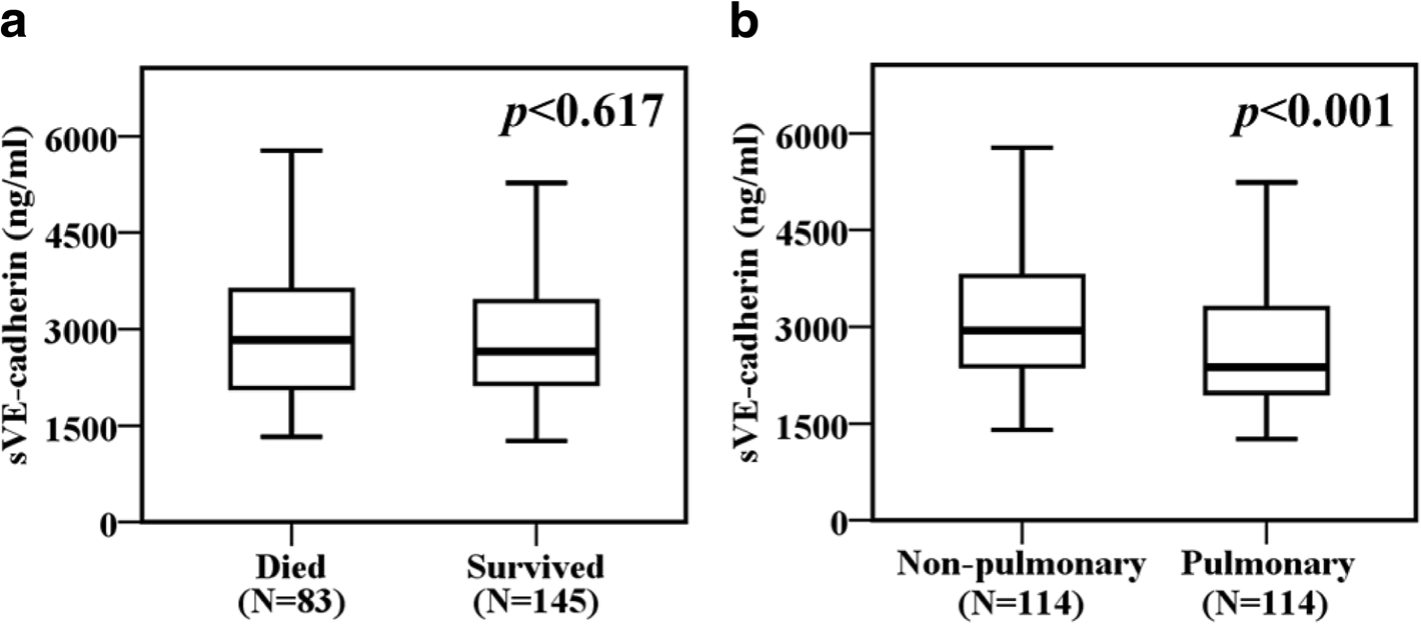
**a** There was no significant difference in plasma sVE-cadherin levels between patients who died or survived from severe sepsis. **b** Plasma sVE-cadherin levels were significantly higher in patients with non-pulmonary sepsis compared to those with pulmonary sepsis. Data were summarized as boxplots where box encompasses 25th–75th percentile, error bars encompass 10th–90th percentile, and horizontal line shows median, and groups were compared by Mann-Whitney *U* test

Plasma sVE-cadherin levels in patients who died from severe sepsis were not significantly different from levels in patients who survived (2830 ng/ml, IQR 2070–3628 ng/ml vs. 2649 ng/ml, IQR 2140–3440 ng/ml, *p* = 0.617, Fig. 3a). Plasma sVE-cadherin levels were higher in non-pulmonary sepsis patients compared with pulmonary sepsis patients (2942 ng/ml, IQR 2384–3796 ng/ml vs. 2367 ng/ml, IQR 1958–3305 ng/ml, *p* < 0.001, Fig. 3b).

## Discussion

Injury and dysfunction of the microvascular endothelium, the pathophysiologic hallmarks of sepsis, lead to shedding of VE-cadherin from the endothelium into the circulation. The degree of shedding of VE-cadherin is correlated with the severity of disease in sepsis by APACHE II score [19]. In the present study, we found that sVE-cadherin levels are elevated in sepsis patients with more organ failures and are associated with AKI-RRT. These results suggest that disruption of endothelial adherens junctions is a prominent feature of sepsis-induced severe AKI.

Sepsis commonly leads to organ dysfunction or failure, with AKI being one of the most common sepsis-related organ dysfunctions. Decreased renal blood flow in sepsis results in endothelial activation and increased renal microvascular permeability [24, 35]. Several studies have investigated the mechanisms underlying increased microvascular permeability in sepsis. Flemming et al. found that treating human dermal microvascular endothelial cells with tumor necrosis factor-α (TNF-α) or bacterial lipopolysaccharide (LPS) caused VE-cadherin shedding from the endothelium and increased sVE-cadherin levels in the cell culture supernatant [17]. In a mouse model of intraperitoneal LPS injection, VE-cadherin mRNA levels in the kidney increased at the 24th hour after LPS injection [36], but the expression of VE-cadherin on renal endothelium was not changed significantly. Herwig et al. reported that the expression of VE-cadherin on pulmonary endothelium was decreased in septic ARDS patients compared with patients receiving lobectomy for lung malignancy [37]. Furthermore, Carden et al. reported that the plasma levels of sVE-cadherin were elevated in ARDS patients compared with healthy subjects [13]. Taken together, these studies suggest that shedding of VE-cadherin may exceed the production of VE-cadherin in sepsis, leading to disruption of adherens junctions and increased endothelial permeability. In a small study of 38 consecutive patients with and without sepsis, Zhang et al. reported that sVE-cadherin levels were elevated in sepsis patients and were associated with mortality [19]. We extend these findings in the present study of 228 subjects with sepsis to study the association between organ dysfunction and sVE-cadherin with a specific focus on acute kidney injury. In the present study, to enrich for patients likely to develop AKI-RRT, we only enrolled severe sepsis patients with APACHE II score greater than 25 and respiratory failure requiring mechanical ventilation. We found that the plasma levels of sVE-cadherin, as an indicator of endothelial injury, were increased in sepsis patients with severe AKI. Furthermore, AKI-RRT was correlated with the level of sVE-cadherin even after adjusting for other AKI risk factors. A rise in sVE-cadherin could be a biomarker for early detection of severe AKI, but larger confirmatory studies are needed. To our knowledge, this is the first study of sVE-cadherin as a biomarker for sepsis patients with severe AKI.

Injury and dysfunction of the liver endothelium may also result in shedding of VE-cadherin into the circulation. In a mouse hemorrhagic shock model, mRNA levels of VE-cadherin in liver cryoresections were elevated within 90 min and persisted after resuscitation [38]. Using a biochip-based, organoid model of human liver sinusoid with human umbilical vascular endothelial cells, monocytes and hepatocytes, Groger et al. found that the expression of VE-cadherin in the liver endothelium and MRP-2, a biliary transport protein, in hepatocytes was decreased after LPS stimulation [39]. In the current study, we found that the plasma levels of sVE-cadherin were elevated in sepsis patients with liver dysfunction. Taken together, these studies suggest that shedding of VE-cadherin may also exceed the production of VE-cadherin in injured liver endothelium.

Aslan et al. measured VE-cadherin expression in a mouse model with intraperitoneal injection of LPS and reported discrepancies in the degree of VE-cadherin expression between the lung and the kidney [36]. The mRNA levels of VE-cadherin in kidney were decreased at the 4th and 8th hour but increased at the 24th hour after LPS injection. The mRNA levels of VE-cadherin in the lung were decreased at the 24th hour. For VE-cadherin protein expression, there was no significant difference in kidney within 24 h. But there was a decrease in the protein expression of VE-cadherin in the lung at the 24th hour. Beyond these data, little is known about the degree of VE-cadherin shedding from the endothelium in different organs or species and this is an important topic for future studies.

Sepsis is a heterogeneous clinical syndrome that can be caused by a variety of underlying infections. Calfee et al. reported that biomarkers of endothelial injury, including angiopoietin-2, were higher in patients with ARDS due to non-pulmonary sepsis than ARDS due to pulmonary sepsis [40]. Murphy et al. reported greater elevation of plasma syndecan-1, a degradation product of the endothelial glycocalyx, in ARDS patients with non-pulmonary sepsis compared to those with pulmonary sepsis [27]. The current study is concordant with the prior reports, demonstrating that plasma sVE-cadherin levels were higher in non-pulmonary sepsis patients compared with pulmonary sepsis patients. Taken together, these studies provide a robust body of evidence that clinical non-pulmonary sepsis is characterized by more severe endothelial injury than pulmonary sepsis. Furthermore, Zhang et al. reported that the levels of sVE-cadherin were correlated with the severity of sepsis [19]. Our study builds on that finding, showing that sepsis patients with higher plasma levels of sVE-cadherin had greater numbers of dysfunctional organs. Profound and widespread endothelial injury may be a potential contributor to the number and severity of organ failures. The role of endothelial injury in the distinct molecular phenotypes of pulmonary and non-pulmonary sepsis and the association between the degree of endothelial injury and the severity of organ dysfunction in sepsis could have important implications for the development of sepsis therapies that target endothelial injury.

Our study has some limitations. First, this was an observational, single-center study. Measurement of plasma sVE-cadherin in a larger multi-center study should be performed to validate these results. Second, we only evaluated the sVE-cadherin level at enrollment on the morning of ICU day 2; whether the change of plasma sVE-cadherin over time is in accordance with the disease progression is not known. Third, in order to enrich for more severely ill patients, this study enrolled only mechanically ventilated sepsis patients with an APACHE II score greater than 25. For this reason, these findings cannot be generalized to other less severely ill sepsis patients, patients without respiratory failure, or critically ill patients without sepsis. The high severity of illness in this cohort may be one reason that we did not detect an association between higher sVE-cadherin and mortality. Fourth, we could not directly measure the integrity of adherens junctions in our study, so it is not possible to determine whether the elevated levels of sVE-cadherin in this study are reflective of decreased adherens junction integrity in vivo. In our study, the plasma levels of sVE-cadherin were not associated with fluid accumulation during the study period. Whether other biomarkers of increased vascular permeability are associated with shedding of sVE-cadherin is unknown. Finally, little is known about the mechanisms of clearance of plasma sVE-cadherin. Doulgere et al. reported plasma sVE-cadherin levels were still elevated when serum creatinine levels decreased or were close to normal range in patients with hemolytic uremic syndrome due to Shiga toxin 2 producing *Escherichia coli* infection (STEC-HUS) [41]. Ebihara et al. reported that there was no difference in plasma sVE-cadherin levels before and after renal replacement therapy in sepsis patients [42]. Whether elevated levels of sVE-cadherin are in part due to impaired clearance in the setting of other organ dysfunction needs to be further investigated.

## Conclusion

In conclusion, the present study demonstrates that plasma sVE-cadherin is independently associated with severe AKI requiring renal replacement therapy in patients with severe sepsis. sVE-cadherin levels are also associated with hepatic failure and the overall number of organ dysfunctions. Patients with non-pulmonary sepsis had higher sVE-cadherin levels compared to those with pulmonary sepsis, suggesting that endothelial injury is more prominent in non-pulmonary sepsis. Further studies of sVE-cadherin as a biomarker of AKI severity in sepsis are needed in larger patient cohorts to better elucidate the role of VE-cadherin shedding in sepsis-induced severe AKI.

## Abbreviations

ADAM: A disintegrin and metalloprotenase
AKI: Acute kidney injury
AKI-RRT: Acute kidney injury requiring renal replacement therapy
APACHE II: Acute physiology and chronic health evaluation II
ARDS: Acute respiratory distress syndrome
ICU: Intensive care unit
IQR: Interquartile range
KDIGO: Kidney Disease Improving Global Outcomes
LPS: Lipopolysaccharide
sVE-cadherin: Soluble vascular endothelial cadherin
TNF-α: Tumor necrosis factor-α
VALID: Validating Acute Lung Injury markers for Diagnosis
VE-cadherin: Vascular endothelial cadherin
